# Molecular Investigation of miRNA Biomarkers as Chemoresistance Regulators in Melanoma: A Protocol for Systematic Review and Meta-Analysis

**DOI:** 10.3390/genes13010115

**Published:** 2022-01-08

**Authors:** Peter Shaw, Greg Raymond, Katherine S. Tzou, Siddhartha Baxi, Ravishankar Ram Mani, Suresh Kumar Govind, Harish C. Chandramoorthy, Palanisamy Sivanandy, Mogana Rajagopal, Suja Samiappan, Sunil Krishnan, Rama Jayaraj

**Affiliations:** 1 Department of Artificial Intelligence, Nanjing University of Information Science and Technology (NUIST), Nanjing 211544, China; 100001@nuist.edu.cn; 2 Menzies School of Health Research, Darwin 0810, Australia; 3 Northern Territory Medical Program, CDU Campus, Flinders University, Ellengowan Drive, Darwin 0909, Australia; greg.raymond@flinders.edu.au; 4 Department of Radiation Oncology, Mayo Clinic Florida, 4500 San Pablo Road S, Jacksonville, FL 32224, USA; tzou.katherine@mayo.edu (K.S.T.); Krishnan.Sunil@mayo.edu (S.K.); 5 Genesis Care Gold Coast Radiation Oncologist, John Flynn Hospital, 42 Inland Drive, Tugun 4224, Australia; Siddhartha.baxi@genesiscare.com; 6 Department of Pharmaceutical Biology, Faculty of Pharmaceutical Sciences, UCSI University Kuala Lumpur (South Wing), No.1, Jalan Menara Gading, UCSI Heights, Cheras, Kuala Lumpur 56000, Malaysia; Ravishankar@ucsiuniversity.edu.my; 7 Department of Parasitology, Faculty of Medicine, University of Malaya, Kuala Lumpur 50603, Malaysia; suresh@um.edu.my; 8 Stem Cells and Regenerative Medicine Unit, Department of Microbiology and Clinical Parasitology, College of Medicine, King Khalid University, Abha 61421, Saudi Arabia; hshkonda@kku.edu.sa; 9 Department of Pharmacy Practice, School of Pharmacy, International Medical University, Bukit Jalil, Kuala Lumpur 57000, Malaysia; PalanisamySivanandy@imu.edu.my; 10 Faculty of Pharmaceutical Sciences, UCSI University Kuala Lumpur (South Wing), No.1, Jalan Menara Gading, UCSI Heights, Cheras, Kuala Lumpur 56000, Malaysia; mogana@ucsiuniversity.edu.my; 11 Department of Biochemistry, Bharathiar University, Coimbatore 641046, India; sujaramalingam08@gmail.com; 12 Northern Territory Institute of Research and Training, Darwin 0909, Australia

**Keywords:** chemoresistance, chemosensitivity, melanoma, meta-analysis, miRNAs, protocol, systematic review

## Abstract

Introduction: Melanoma is a global disease that is predominant in Western countries. However, reliable data resources and comprehensive studies on the theragnostic efficiency of miRNAs in melanoma are scarce. Hence, a decisive study or comprehensive review is required to collate the evidence for profiling miRNAs as a theragnostic marker. This protocol details a comprehensive systematic review and meta-analysis on the impact of miRNAs on chemoresistance and their association with theragnosis in melanoma. Methods and analysis: The articles will be retrieved from online bibliographic databases, including Cochrane Review, EMBASE, MEDLINE, PubMed, Scopus, Science Direct, and Web of Science, with different permutations of ‘keywords’. To obtain full-text papers of relevant research, a stated search method will be used, along with selection criteria. The Preferred Reporting Items for Systematic Reviews and Meta-Analysis for Protocols 2015 (PRISMA-P) standards were used to create this study protocol. The hazard ratio (HR) with a 95% confidence interval will be analyzed using Comprehensive Meta-Analysis (CMA) software 3.0. (CI). The pooled effect size will be calculated using a random or fixed-effects meta-analysis model. Cochran’s Q test and the I2 statistic will be used to determine heterogeneity. Egger’s bias indicator test, Orwin’s and the classic fail-safe N tests, the Begg and Mazumdar rank collection test, and Duval and Tweedie’s trim and fill calculation will all be used to determine publication bias. The overall standard deviation will be evaluated using Z-statistics. Subgroup analyses will be performed according to the melanoma participants’ clinicopathological and biological characteristics and methodological factors if sufficient studies and retrieved data are identified and available. The source of heterogeneity will be assessed using a meta-regression analysis. A pairwise matrix could be developed using either a pairwise correlation or expression associations of miRNA with patients’ survival for the same studies.

## 1. Introduction

### 1.1. Epidemiology

Skin cancers are uncommon malignancies globally and do not rank among the top ten common cancers [1]. Despite melanoma not being the leading cause of cancer mediated deaths, deaths from melanoma are rising, and it has a vastly inferior prognosis compared to other common types of cancer. The three primary types of skin cancer are basal cell carcinoma, squamous cell carcinoma, and malignant melanoma. Squamous and basal cell carcinoma together are referred to as non-melanoma skin cancers [2]. Among the known skin cancers, the most commonly occurring type is basal cell carcinoma [3]. Despite the fact the global incidence of melanoma (1.6 percent) is lower than that of non-melanoma skin cancers (6.2 percent), melanoma is still considered a progressive disease and is the deadliest form of skin cancer [4]. About 75% of the skin-cancer associated deaths are due to melanoma [5]. It is a rare type of skin cancer that progresses to other parts of the body. Its risk factors include exposure of skin to ultraviolet (UV) light and other factors, such as genetics [6,7]. Although surgery is the standard treatment after diagnosis, prevention strategies are prioritized, as the best way to avoid developing melanoma is by limiting direct exposure to sunlight and, therefore, overexposure to UV light [8].

### 1.2. Rationale

#### 1.2.1. The Importance of This Study

Chemoresistance continues to be a significant impediment to cancer treatment in medical oncology. Resistance may occur due to prior exposure or even as a result of cancer therapy itself [9,10]. Thus, research on treatment strategies, such as the multimodality approach involving surgery, chemotherapy, radiotherapy, and immune/biotherapy, is currently being conducted in order to circumvent the issue of the development of chemoresistance [11]. miRNAs’ involvement in melanoma chemoresistance has not been effectively explored [12,13,14]. Recent and emerging studies on this topic have generated sufficient clinical data to make a more feasible approach to perform a meta-analysis and systematic review on melanoma patients’ chemoresistance [15,16,17].

#### 1.2.2. What Will the Study’s Approach Be to This Problem?

The suggested study has the potential to provide a comprehensive picture of chemo-resistance in melanoma and its relationship to miRNA expression. Non-coding RNAs called microRNAs influence gene expression. miRNAs are small RNAs with a length of 19–25 nucleotides that inhibit or degrade genes at the post-transcriptional phase [18]. Several studies have focused on miRNAs’ impact on chemotherapeutic resistance in cancers, including breast cancer [19], cervical cancer [20], colorectal cancer [21], gastric cancer [22], lung cancer [23], oral cancer [24], ovarian cancer [25], pancreatic cancer [26] prostate cancer [27], and skin cancer [28]. With a 5-year survival rate of 92 percent, surgery remains the best choice for curing localized, invasive melanoma [29]. The molecular basis of melanoma resistance to chemotherapy is thought to be multifactorial, with a defective drug transport system, an altered apoptotic pathway, apoptosis deregulation, and changes in the enzymatic systems that mediate cellular metabolic machinery all contributing to chemotherapy complications [30].

There have also been several meta-analyses and systematic reviews considering the link between miRNAs and chemoresistance [31,32]. However, topics, such as the clinical outcome predictions of miRNAs in cancer [33], the miRNA prognostic signatures cross-validated in metastatic melanoma [34], and the correlation between DNA repair gene polymorphism and cutaneous melanoma, still require further investigation [35]. Understanding the impact of changes in chemoresistance-related biological processes could aid in the development of new therapeutic approaches for malignant melanoma treatment [36].

#### 1.2.3. How Will It Help?

The role played by microRNAs in chemoresistance is found to be complex, and linking distinct miRNAs to different genetic pathways is still in its infancy. Clinical samples can benefit from miRNA profiling and can allow for the distinguishing of cancerous cells from normal cells and could be a useful tool for classifying poorly differentiated tumors. Providing a detailed systematic review may aid oncologists, gastroenterologists, and clinical researchers to expand their understanding of the theragnostic and predictive role of miRNAs and the potential implementation of these biomarkers for future clinical practice. Therefore, studies analyzing the effects of miRNA expression on chemoresistance and sensitivity in melanoma patients, as well as studies exploring the effects of miRNAs on chemotherapy via in vitro experimentation, will be included in our review. Our study will provide a network of chemoresistance mechanisms and drug regulatory pathways in conjunction with the different chemotherapy drugs commonly utilized in melanoma. Thus, it is hoped that identifying the specific miRNAs and the associated pathways of chemoresistance in melanoma may help in the development of future therapeutics by indicating how miRNAs’ profiles could predict the efficacy of chemotherapy and chemoresistance. The results obtained from the meta-analysis will ideally help improve clinical treatment and prognosis [37,38]. This protocol and the study following it should act as a reference for future studies regarding the prognosis and diagnosis of melanoma using microRNAs and, thereby, help in the proliferation of literature in this field.

## 2. Methods

### 2.1. Study Design

An all-encompassing search approach will be carried out using the bibliographic databases Cochrane Review, MEDLINE, EMBASE, PubMed, Science Direct, Scopus, and Web of Science for the last ten years. Previous studies evaluating the role of miRNAs on chemoresistance and sensitivity in melanoma will be identified. Additional studies will be extracted from the included studies’ reference lists through a manual search and also from the review of literature articles that discuss melanoma chemoresistance. The publication language will be limited to English, including officially translated materials, with no restrictions on the publication date or status.

#### 2.1.1. Eligibility Criteria

##### Inclusion Criteria

As a key inclusion criterion, investigations must examine at the impact of miRNA expression in melanoma patients and cell lines.

Other norms will include:

- Studies that deal with resistance in melanoma.
- Studies published until December 2021.
- Reporting of miRNA profiling platforms.
- Studies with appropriate patient data with therapeutic measures.
- Studies reporting the genes and/or pathways involved in chemoresistance or chemosensitivity.
- miRNA expression analysis using in vitro assays.
- Studies reporting the patient’s survival with 95% CI (confidence interval) values in hazard ratio (HR) or Kaplan–Meier (KM) curves for quantitative synthesis or meta-analysis.

##### Exclusion Criteria

The following will be excluded from the study:

- Letters to the editor, fact sheets, conference proceedings, unpublished materials, review articles, case studies, and studies conducted solely in patients or in vitro.
- Studies examining patient data from bioinformatic datasets.
- Duplicate publications from the same study will be treated as one study.
- Studies using non-human data.

##### Search Strategy and Study Selection

Databases will be used to identify the literature that is related to miRNAs, drug resistance, and melanoma. The search terms used should be in all combinations of “miRNA” or “microRNA” AND “Drug resistance” or “Chemosensitivity” or “Chemoresistance” AND “Melanoma” (Table 1). Additional relevant articles will be identified by manually examining the retrieved articles. Potentially relevant articles will be carefully collated for further processing. The studies will initially be chosen based on the individual judgement of two authors upon the reading of the titles and abstracts of the articles. Full-text articles will be scrutinized if the titles and abstracts are uncertain. All authors will be contacted for pertinent information. Any disagreement will be solved by discussion amongst the two authors. Any major differences will involve a team decision or third reviewer to make a decision.

##### Data Extraction and Management

The studies in the selection criteria will be evaluated individually and the respective authors will be contacted by the authors. to gather any missing information. The data extraction form will collect bibliographic and demographic information, as well as clinicopathological and biological aspects of melanoma patients if relevant data and information are available. Data from the included studies will be reviewed by three authors and cross-checked by the corresponding author. The corresponding authors of the selected articles will be contacted for further clarifications.

#### 2.1.2. Data Collection Process

1. From the studies, five major categories of data will be extracted: The study characteristics, including the author, geographic region, year of publication, study period, sample size, study design, sampling procedures, validity of confirmative diagnosis, method of data collection, and number of melanoma cancer cases/patients, as well as the International Classification of Disease (ICD) Code for the anatomical site of cancer under study.
2. Clinical, pathological, and biological attributes, including comorbidity, risk factors, tumor histology (squamous, adenocarcinoma, clear cell, and undifferentiated), pathological grades (1, 2, and 3), tumor size, negative and positive lymph node metastasis, positive and negative vascular involvement, the lymphocyte infiltration (if any), histology grade (well, moderate, poor, and undetermined), P16 (positive and negative), deep stromal invasion (%), and specific body sites, such as the face (the temporal, frontal, periorbital, infraorbital, buccal, zygomatic, mental, or perioral region), nose, lip, ear, scalp, trunk, neck, and extremities [39].
3. miRNA expression in melanoma patients and their responses towards their treatment.
4. Hazard ratio (HR) and 95% confidence interval (CI) estimates of overall survival (OS), disease-free survival (DFS), and other endpoint measures.
5. In vitro and in vivo studies.

##### Outcomes and Prioritization

The primary outcome is to evaluate the role of the miRNAs associated with chemoresistance in melanoma patients.

Secondary outcomes will be used to correlate variations in primary outcomes with clinicopathological and biological parameters.

##### Quality Assessment of Included Studies

The Dutch Cochrane Centre’s Meta-Analysis Of Observational Studies in Epidemiology (MOOSE) guidelines [40] will be used to assess the quality of the included studies, and the following information will be extracted:

i. Information about the patient’s tissue collection.
ii. Location of the study.
iii. Gender.
iv. Age.
v. Exposure to sunlight.
vi. Ulceration status.
vii. miRNA analysis in melanoma patients.
viii. List of melanoma cell lines used.
ix. Tumor stage.
x. Lymph node status.
xi. miRNA profiling platform.
xii. The form of therapy used.
xiii. Genes and/or pathways involved in resistance.

All the mentioned criteria will be required for the study to be qualified for the systematic review. The Newcastle-Ottawa scale (NOS) will also be used to assess the methodological quality of cohort studies [41].

##### Assessment of Risk of Bias in Individual Studies

The authors will assess the risk of bias based on parameters, such as the number of patients studied, the year of publication, the mode of disease diagnosis, geographical demarcation, and the length of the study. A predetermined checklist incorporating questions from eight categories from the Dutch Cochrane Centre’s Meta-Analysis Of Observational Studies in Epidemiology (MOOSE) guidelines will be used to assess the quality of the studies [40]. Six elements will be included in the tool’s reporting: background, search strategy, techniques, results, discussion, and conclusions. The reporting elements of the checklist are based on epidemiological concepts and will be provided even if individual studies lack strong empirical evidence [42,43,44,45].

##### Publication Bias

A significant concern in meta-analysis is the risk of publication bias [46,47,48,49,50]. To understand publication bias, Egger’s and Begg’s bias indicator tests, as well as the inverted funnel plot, will be used [51]. Trim and fill calculations by Duval and Tweedie will also be evaluated [52]. To investigate the effect size of statistically non-significant and unpublished studies, the classic and Orwin’s fail-safe N tests will be used [53,54,55,56,57,58]. All the authors will assess publication bias individually. Team decisions will be involved in case of disagreements.

#### 2.1.3. Statistical Analysis

##### Meta-Analysis

Meta-regression analysis will be used to study the heterogeneity between the involved studies. Potential influences, such as the number of patients, year of publication, study period, research location, kind of study, and diagnostic process will be investigated for heterogeneity using the Higgins I-squared statistic [59] and Cochran’s Q test [60].

The hazard ratio (HR) will be analyzed using the comprehensive meta-analysis software (CMA) 3.0 with a 95% CI (confidence interval). Fixed model effects will be used in significant studies and, if studies are not significant, random model effects will be used. Z-statistics will be used to calculate the overall standard deviation.

##### Subgroup Analyses

Subgroup analyses will be performed based on the melanoma participants’ clinical, pathological, and biological characteristics, as well as methodological aspects, if adequate studies and recovered data are discovered and accessible. Our research team intends to look into particular subgroup analyses based on clinical and pathological factors and biological information, such as comorbidity, risk factors, tumor histology (squamous, adenocarcinoma, clear cell, and undifferentiated), pathological grades (1, 2, and 3), tumor size, negative and positive lymph node metastasis, negative and positive vascular involvement, histology grade (well, moderate, poor, and undetermined), P16 (positive and negative), deep stromal invasion (percentage), and specific body sites, such as the face (the temporal, frontal, periorbital, infraorbital, buccal, zygomatic, mental, or perioral region), nose, lip, ear, neck, scalp, trunk, and other parameters.

##### Meta-Regression

A meta-regression analysis will be used to determine the source of heterogeneity. A P-value of less than 0.05 will be considered significant for heterogeneity. Gender distribution, data collection methods, research quality, sample size, and sampling procedure will all be evaluated. In order to weigh every study by calculating R^2^ with the proposed quantity variance, a random-effects model will be employed. A meta-regression analysis will be used to explain the heterogeneity of cancer research in relation to one or more study variables, with a large ratio of studies required for a genuine regression. For each deviation, a ratio of at least 10 is recommended [61,62,63,64].

##### Network-Centric Model Analysis

**Figure 1:**
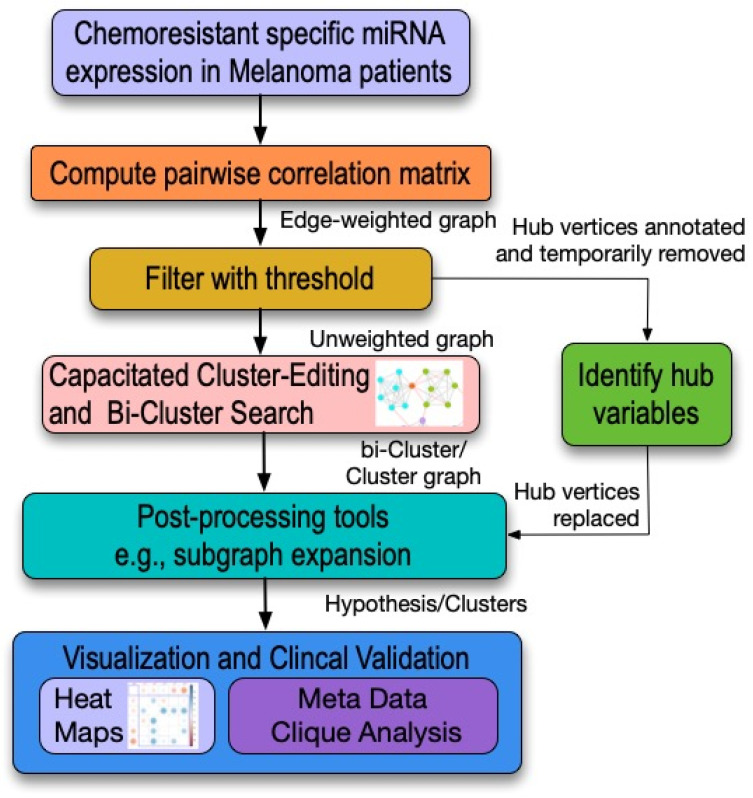
Network analysis toolchain. The output of the produced clustering would consist of interacting RNA or patient symptoms (also known as a generated hypothesis). To confirm and consider this hypothesis, a systematic review or meta-data search for papers that may already have considered two or more of the factors listed in these clusters should be carried out.

A pairwise matrix could be developed using either pairwise correlation or expression associations of miRNA with patients’ survival for the same papers listed in this protocol [65,66]. A clique-centric pattern search using cluster editing could then be used to identify pathways/systems [67,68]. The details of this analysis toolkit are summarized in Figure 1. The strength of this approach stems from the fact that that it provides more insight into the upregulated and downregulated expression of the miRNAs and the melanoma cancers that are not possible with other statistical methods such as principle component analysis.

##### Random Forest Analysis

A random forest analysis provides a robust means of feature selection of miRNA expression. The results of this analysis can then be used to develop prognostic value tools, such as decision trees. By coupling a random forest analysis with the other ensemble methods, such as those provided by the R interface for ‘H2O’(R H2O package), the scalable open source machine learning platform, artificial intelligence (AI) prognosis tools could also be produced [69]. Nevertheless, a random forest analysis is also an effective way of identifying robust features for predictive modelling. Alternatively, a principal component analysis (PCA) and a clustering algorithm (such as k-means) can be used. However, these techniques work, as long as the number of attributes or dimensions does not exceed five for most of the variation [70,71].

### 2.2. Presenting and Reporting the Review Results

**Figure 2:**
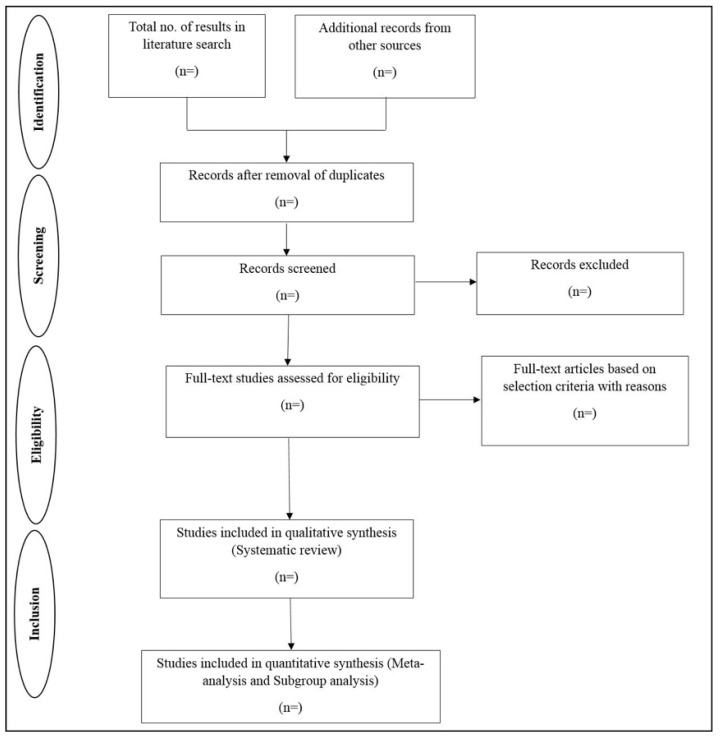
Flowchart for the systematic review.

This protocol was written in accordance with the PRISMA-P statement (http://www.prisma-statement.org/Extensions/Protocols; accessed on 9 September 2021) [72]. The findings will be made public in accordance with the PRISMA criteria [73]. A flowchart outlining the selection process (to be used) is shown in Figure 2. The included studies’ qualitative data will be evaluated descriptively. A forest plot will be used to depict the outputs of the meta-analyses. An inverted funnel plot will be used to represent publication bias, based on Egger’s graphical test for publication bias. The search strategy, PRISMA-P checklist, and the quality appraisal tool will be made available as a supplement.

### 2.3. Ethics and Dissemination

Because this study will not include human subjects, it will not require a formal human research ethical review or approval from a human research ethics committee. It will be carried out with publicly available anonymized data and will not need formal human study, ethical review, or permission from a human research ethics board. We want to distribute our findings by publishing them in peer-reviewed publications and discuss them in relevant conference proceedings. We also expect that the systematic review’s findings will have ramifications for policy and clinical practice. We will create a policymaker-friendly summary in a validated style, which we will share via social media and email discussion groups.

### 2.4. Strengths and Limitations of This Study

This protocol will help researchers carry out systematic reviews and meta-analyses of the randomized data obtained from various research studies.

- PRISMA-P (Preferred Reporting Items for Systematic Reviews and Meta-Analyses Protocol) recommendations are followed in the protocol.
- It will help researchers make informed decisions, due to specific evidence obtained from organized data.
- This study will help us obtain a clear picture of the role of miRNAs on chemoresistance for melanoma patients.
- Certain forms of data obtained from various literature may be challenging to incorporate due to statistical error and, hence, may hamper the outcome.

## 3. Discussion

Previous research has found a relationship between miRNA expression and melanoma prognosis; however, little is known about miRNAs’ prognostic value in melanoma.

There has never been a thorough investigation or meta-analysis of the function of miRNA in chemoresistance melanoma. The statistical accounts of the risk factors associated with this disease can only be uncovered by investigating more studies associated with miRNA expression in melanoma patients. The studies analyzed through this protocol can help determine the relationship between chemoresistance and patient survival. Usually, the clinical studies reported are confined to a limited population, over a short period. Hence, this protocol for systematic review and meta-analysis could provide an organized overview of the role of chemoresistance-specific miRNA expression in melanoma. The results obtained using this protocol will aid the physician’s ability to make an informed decision and would result in a better quality of life for melanoma patients.

This study could provide reliable and productive results which may help in further research. The proposed protocol would build upon available studies highlighting the significance of miRNAs in effecting chemoresistance and sensitivity. Any extrapolations, unless specified in the protocol, are not recommended.

## Figures and Tables

**Table 1:** Search terms.

Search Number	Parameter
1	Melanoma “[Topic]” OR miRNA “[Topic]”
2	Melanoma “[Topic]” OR miRNA “[Topic]” OR patient “[Topic]” OR clinical study “[Topic]”
3	Melanoma “[Topic]” OR miRNA “[Topic]” OR microRNA “[Topic]” AND resistance “[Topic]” OR patient “[Topic]” OR clinical study “[Topic]”
4	Melanoma “[Topic]” OR miRNA “[Topic]” OR microRNA “[Topic]” AND chemoresistance (Chemoresist*) “[Topic]” OR patient “[Topic]” OR clinical study “[Topic]”
5	Melanoma “[Topic]” OR miRNA “[Topic]” OR microRNA “[Topic]” AND chemosensitivity (Chemosens*) “[Topic]” OR patient “[Topic]” OR clinical study “[Topic]”
6	1 AND 2 AND 3 AND 4 AND 5

* The search terms “Chemosensitivity” or “Chemoresistance” will be substituted by wildcards, such as ”Chemosens*“ or “Chemoresist*”.

## Data Availability

Not applicable.

## Abbreviations

miRNA: MicroRNA; BCC: basal cell carcinoma; SCC: squamous cell carcinoma; R interface for ‘H2O’(R H2O package), artificial intelligence (AI), principal component analysis (PCA), a clustering algorithm (such as k-means), HR: hazard ratio; CI: confidence interval; NOS: overall survival (OS), Disease-free survival (DFS) and other endpoint measures Newcastle-Ottawa scale; PRISMA: Preferred Reporting Items for Systematic Review and Meta-analysis; PRISMA-P: Preferred Reporting Items for Systematic Review and Meta-analysis protocol; ICD: International Classification of Disease; CMA: Comprehensive Meta-Analysis.

## References

[1] Ferlay J. (2001). GLOBOCAN 2000. Cancer Incidence, Mortality and Prevalence Worldwide.

[2] Franceschi S., Levi F., Randimbison L., La Vecchia C. (1996). Site distribution of different types of skin cancer: New aetiological clues. Int. J. Cancer.

[3] Godbole V., Toprani H., Shah H. (1968). Skin cancer in Saurashtra. Indian J. Pathol. Bacteriol..

[4] Sung H., Ferlay J., Siegel R.L., Laversanne M., Soerjomataram I., Jemal A., Bray F. (2021). Global cancer statistics 2020: GLOBOCAN estimates of incidence and mortality worldwide for 36 cancers in 185 countries. CA A Cancer J. Clin..

[5] Chang D.T., Amdur R.J., Morris C.G., Mendenhall W.M. (2006). Adjuvant radiotherapy for cutaneous melanoma: Comparing hypofractionation to conventional fractionation. Int. J. Radiat. Oncol. Biol. Phys..

[6] Armstrong B.K., Kricker A. (2001). The epidemiology of UV induced skin cancer. J. Photochem. Photobiol. B Biol..

[7] Haluska F.G., Hodi F.S. (1998). Molecular genetics of familial cutaneous melanoma. J. Clin. Oncol..

[8] Koh H.K., Kligler B.E., Lew R.A. (1990). Sunlight and cutaneous malignant melanoma: Evidence for and against causation. Photochem. Photobiol..

[9] Kerbel R.S. (1997). A cancer therapy resistant to resistance. Nature.

[10] Wagle N., Emery C., Berger M.F., Davis M.J., Sawyer A., Pochanard P., Kehoe S.M., Johannessen C.M., MacConaill L.E., Hahn W.C. (2011). Dissecting therapeutic resistance to RAF inhibition in melanoma by tumor genomic profiling. J. Clin. Oncol..

[11] Sharma K., Mohanti B.K., Rath G.K. (2009). Malignant melanoma: A retrospective series from a regional cancer center in India. J. Cancer Res. Ther..

[12] Müller D., Bosserhoff A. (2008). Integrin β 3 expression is regulated by let-7a miRNA in malignant melanoma. Oncogene.

[13] Dar A.A., Majid S., de Semir D., Nosrati M., Bezrookove V., Kashani-Sabet M. (2011). miRNA-205 suppresses melanoma cell proliferation and induces senescence via regulation of E2F1 protein. J. Biol. Chem..

[14] Pencheva N., Tran H., Buss C., Huh D., Drobnjak M., Busam K., Tavazoie S.F. (2012). Convergent multi-miRNA targeting of ApoE drives LRP1/LRP8-dependent melanoma metastasis and angiogenesis. Cell.

[15] Madhav M.R., Nayagam S.G., Biyani K., Pandey V., Kamal D.G., Sabarimurugan S., Ramesh N., Gothandam K.M., Jayaraj R. (2018). Epidemiologic analysis of breast cancer incidence, prevalence, and mortality in India: Protocol for a systematic review and meta-analyses. Medicine (Baltimore).

[16] Poddar A., Aranha R.R., Muthukaliannan G.K., Nachimuthu R., Jayaraj R. (2018). Head and neck cancer risk factors in India: Protocol for systematic review and meta-analysis. BMJ Open.

[17] Jayaraj R., Kumarasamy C., Piedrafita D. (2018). Systematic review and meta-analysis protocol for Fasciola DNA vaccines. Online J. Vet. Res..

[18] Kim W.T., Kim W.-J. (2013). MicroRNAs in prostate cancer. Prostate Int..

[19] Lin Teoh S., Das S. (2017). The role of MicroRNAs in diagnosis, prognosis, metastasis and resistant cases in breast cancer. Curr. Pharm. Des..

[20] Zhang W., Zhou J., Zhu X., Yuan H. (2017). MiR-126 reverses drug resistance to TRAIL through inhibiting the expression of c-FLIP in cervical cancer. Gene.

[21] Zhang Y., Wang J. (2017). MicroRNAs are important regulators of drug resistance in colorectal cancer. Biol. Chem..

[22] Yang W., Ma J., Zhou W., Cao B., Zhou X., Yang Z., Zhang H., Zhao Q., Fan D., Hong L. (2017). Molecular mechanisms and theranostic potential of miRNAs in drug resistance of gastric cancer. Expert Opin. Ther. Targets.

[23] Haefliger S., Hudson A., Hayes S., Pavlakis N., Howell V. (2017). P2. 01-012 Acquired Chemotherapy Resistance in vitro: miRNA Profiles of Chemotherapy Resistant Squamous Lung Cancer Cell Lines: Topic: Analysis of RNA. J. Thorac. Oncol..

[24] Zhuang Z., Hu F., Hu J., Wang C., Hou J., Yu Z., Wang T.T., Liu X., Huang H. (2017). MicroRNA-218 promotes cisplatin resistance in oral cancer via the PPP2R5A/Wnt signaling pathway. Oncol. Rep..

[25] Tung S.L., Huang W.C., Hsu F.C., Yang Z.P., Jang T.H., Chang J.W., Chuang C.M., Lai C.R., Wang L.H. (2017). miRNA-34c-5p inhibits amphiregulin-induced ovarian cancer stemness and drug resistance via downregulation of the AREG-EGFR-ERK pathway. Oncogenesis.

[26] Amponsah P.S., Fan P., Bauer N., Zhao Z., Gladkich J., Fellenberg J., Herr I. (2017). microRNA-210 overexpression inhibits tumor growth and potentially reverses gemcitabine resistance in pancreatic cancer. Cancer Lett..

[27] Armstrong C.M., Liu C., Lou W., Lombard A.P., Evans C.P., Gao A.C. (2017). MicroRNA-181a promotes docetaxel resistance in prostate cancer cells. Prostate.

[28] Fattore L., Sacconi A., Mancini R., Ciliberto G. (2017). MicroRNA-driven deregulation of cytokine expression helps development of drug resistance in metastatic melanoma. Cytokine Growth Factor Rev..

[29] Joyce K.M. (2017). Surgical management of melanoma. Exon Publ..

[30] Grossman D., Altieri D.C. (2001). Drug resistance in melanoma: Mechanisms, apoptosis, and new potential therapeutic targets. Cancer Metastasis Rev..

[31] Royam M.M., Kumarasamy C., Baxi S., Gupta A., Ramesh N., Muthukaliannan G.K., Jayaraj R. (2019). Current Evidence on miRNAs as Potential Theranostic Markers for Detecting Chemoresistance in Colorectal Cancer: A Systematic Review and Meta-Analysis of Preclinical and Clinical Studies. Mol. Diagn. Ther..

[32] Wang W., Li J., Zhu W., Gao C., Jiang R., Li W., Hu Q., Zhang B. (2014). MicroRNA-21 and the clinical outcomes of various carcinomas: A systematic review and meta-analysis. BMC Cancer.

[33] Nair V.S., Maeda L.S., Ioannidis J.P. (2012). Clinical outcome prediction by microRNAs in human cancer: A systematic review. J. Natl. Cancer Inst..

[34] Jayawardana K., Schramm S.J., Tembe V., Mueller S., Thompson J.F., Scolyer R.A., Mann G.J., Yang J. (2016). Identification, review, and systematic cross-validation of microRNA prognostic signatures in metastatic melanoma. J. Investig. Dermatol..

[35] Mocellin S., Verdi D., Nitti D. (2009). DNA repair gene polymorphisms and risk of cutaneous melanoma: A systematic review and meta-analysis. Carcinogenesis.

[36] Kalal B.S., Upadhya D., Pai V.R. (2017). Chemotherapy resistance mechanisms in advanced skin cancer. Oncol. Rev..

[37] Lu J., Getz G., Miska E.A., Alvarez-Saavedra E., Lamb J., Peck D., Sweet-Cordero A., Ebert B.L., Mak R.H., Ferrando A.A. (2005). MicroRNA expression profiles classify human cancers. Nature.

[38] Iorio M.V., Visone R., Di Leva G., Donati V., Petrocca F., Casalini P., Taccioli C., Volinia S., Liu C.G., Alder H. (2007). MicroRNA signatures in human ovarian cancer. Cancer Res..

[39] Ceylan C., Oztürk G., Alper S. (2003). Non-Melanoma Skin Cancers between the Years of 1990 and 1999 in Izmir, Turkey: Demographic and Clinicopathological Characteristics. J. Dermatol..

[40] Stroup D.F., Berlin J.A., Morton S.C., Olkin I., Williamson G.D., Rennie D., Moher D., Becker B.J., Sipe T.A., Thacker S.B. (2000). Meta-analysis of observational studies in epidemiology: A proposal for reporting. JAMA.

[41] Wells G., Shea B., O’connell D., Peterson J., Welch V., Losos M., Tugwell P. (2009). The Newcastle-Ottawa Scale (NOS) for Assessing the Quality of Nonrandomised Studies in Meta-Analyses.

[42] Kumarasamy C., Devi A., Jayaraj R. (2018). Prognostic value of microRNAs in head and neck cancers: A systematic review and meta-analysis protocol. Syst Rev..

[43] Jayaraj R., Kumarasamy C. (2018). Systematic review and meta-analysis of cancer studies evaluating diagnostic test accuracy and prognostic values: Approaches to improve clinical interpretation of results. Cancer Manag. Res..

[44] Jayaraj R., Kumarasamy C., Ramalingam S., Devi A. (2018). Systematic review and meta-analysis of risk-reductive dental strategies for medication related osteonecrosis of the jaw among cancer patients: Approaches and strategies. Oral Oncol..

[45] Sabarimurugan S., Royam M.M., Das A., Das S., Gothandam K.M., Jayaraj R. (2018). Systematic Review and Meta-analysis of the Prognostic Significance of miRNAs in Melanoma Patients. Mol. Diagn Ther..

[46] Jayaraj R., Kumarasamy C., Madhav M.R., Pandey V., Sabarimurugan S., Ramesh N., Gothandam K.M., Baxi S. (2018). Systematic Review and Meta-Analysis of Diagnostic Accuracy of miRNAs in Patients with Pancreatic Cancer. Dis. Markers.

[47] Jayaraj R., Kumarasamy C. (2018). Prognostic biomarkers for oral tongue squamous cell carcinoma: A systematic review and meta-analysis. Br. J. Cancer.

[48] Jayaraj R., Kumarasamy C. (2018). Survival for HPV-positive oropharyngeal squamous cell carcinoma with surgical versus non-surgical treatment approach: A systematic review and meta-analysis. J. Oral. Oncol..

[49] Jayaraj R., Kumarasamy C., Sabarimurugan S., Baxi S. (2018). Commentary: Blood-Derived microRNAs for Pancreatic Cancer Diagnosis: A Narrative Review and Meta-Analysis. Front. Physiol..

[50] Jayaraj R., Kumarasamy C. (2019). Conceptual interpretation of analysing and reporting of results on systematic review and meta-analysis of optimal extent of lateral neck dissection for well-differentiated thyroid carcinoma with metastatic lateral neck lymph nodes. Oral Oncol..

[51] Begg C.B., Mazumdar M. (1994). Operating characteristics of a rank correlation test for publication bias. Biometrics.

[52] Duval S., Tweedie R. (2000). Trim and fill: A simple funnel-plot–based method of testing and adjusting for publication bias in meta-analysis. Biometrics.

[53] Orwin R.G. (1983). A fail-safe N for effect size in meta-analysis. J. Educ. Stat..

[54] Jayaraj R., Kumarasamy C., Gothandam K.M. (2018). Letter to the editor "Prognostic value of microRNAs in colorectal cancer: A meta-analysis". Cancer Manag Res..

[55] Jayaraj R., Kumarasamy C. (2018). Letter to the Editor about the Article:" Performance of different imaging techniques in the diagnosis of head and neck cancer mandibular invasion: A systematic review and meta-analysis". J. Oncol..

[56] Jayaraj R., Kumarasamy C., Sabarimurugan S., Baxi S. (2018). Letter to the Editor in response to the article," The epidemiology of oral human papillomavirus infection in healthy populations: A systematic review and meta-analysis". Oral Oncol..

[57] Jayaraj R., Kumarasamy C., Samiappan S., Swaminathan P. (2018). Letter to the Editor regarding, The prognostic role of PD-L1 expression for survival in head and neck squamous cell carcinoma: A systematic review and meta-analysis. Oral Oncol..

[58] Jayaraj R., Kumarasamy C., Madurantakam Royam M., Devi A., Baxi S. (2018). Letter to the editor: Is HIF-1alpha a viable prognostic indicator in OSCC? A critical review of a meta-analysis study. World J. Surg. Oncol..

[59] Higgins J.P., Thompson S.G., Deeks J.J., Altman D.G. (2003). Measuring inconsistency in meta-analyses. BMJ Br. Med. J..

[60] Cochran W.G. (1954). The combination of estimates from different experiments. Biometrics.

[61] Borenstein M., Hedges L.V., Higgins J.P., Rothstein H.R. (2011). Introduction to Meta-Analysis.

[62] Renehan A.G., Zwahlen M., Minder C., T O’Dwyer S., Shalet S.M., Egger M.J.T.L. (2004). Insulin-like growth factor (IGF)-I, IGF binding protein-3, and cancer risk: Systematic review and meta-regression analysis. Lancet.

[63] Sterne J.A., Jüni P., Schulz K.F., Altman D.G., Bartlett C., Egger M. (2002). Statistical methods for assessing the influence of study characteristics on treatment effects in ‘meta-epidemiological’research. Stat. Med..

[64] Thompson S.G., Sharp S.J. (1999). Explaining heterogeneity in meta-analysis: A comparison of methods. Stat. Med..

[65] Ben-Dor A., Shamir R., Yakhini Z. (1999). Clustering gene expression patterns. J. Comput. Biol..

[66] Jay J.J., Eblen J.D., Zhang Y., Benson M., Perkins A.D., Saxton A.M., Voy B.H., Chesler E.J., Langston M.A. (2012). A Systematic Comparison of Genome-Scale Clustering Algorithms.

[67] Abu-Khzam F.N. (2017). On the complexity of multi-parameterized cluster editing. J. Discret. Algorithms.

[68] Dehne F., Langston M.A., Luo X., Pitre S., Shaw P., Zhang Y. (2006). The Cluster Editing Problem: Implementations and Experiments.

[69] Abu-Khzam F.N., Egan J., Gaspers S., Shaw A., Shaw P. (2018). Cluster Editing with Vertex Splitting.

[70] Barr J., Shaw P. (2018). AI Application to Data Analysis, Automatic File Processing.

[71] Landry M., Angela B. (2018). Machine Learning with R and H_2_O.

[72] Shamseer L., Moher D., Clarke M., Ghersi D., Liberati A., Petticrew M., Shekelle P., Stewart L.A. (2015). Preferred reporting items for systematic review and meta-analysis protocols (PRISMA-P) 2015: Elaboration and explanation. BMJ.

[73] Moher D., Liberati A., Tetzlaff J., Altman D.G., Prisma Group (2009). Preferred reporting items for systematic reviews and meta-analyses: The PRISMA statement. PLoS Med..

